# Supplementary material for: A human isogenic iPSC-derived cell line panel identifies major regulators of aberrant astrocyte proliferation in Down syndrome
Source: Commun Biol. 2021 Jun 14;4:730. doi: 10.1038/s42003-021-02242-7 (PMC8203796; doi:10.1038/s42003-021-02242-7)
Supplement: Supplementary file 2 — Supplementary Information [file 42003_2021_2242_MOESM2_ESM.pdf]

Supplementary Information

A human isogenic iPSC-derived cell line panel identifies major regulators of aberrant astrocyte proliferation in Down syndrome

Keiji Kawatani[1], Toshihiko Nambara[1], Nobutoshi Nawa[1], Hidetaka Yoshimatsu[1], Haruna Kusakabe[1], Katsuya Hirata[1,2], Akira Tanave[3], Kenta Sumiyama[3], Kimihiko Banno[1,4], Hidetoshi Taniguchi[1], Hitomi Arahori[1], Keiichi Ozono[1], and Yasuji Kitabatake[1]*

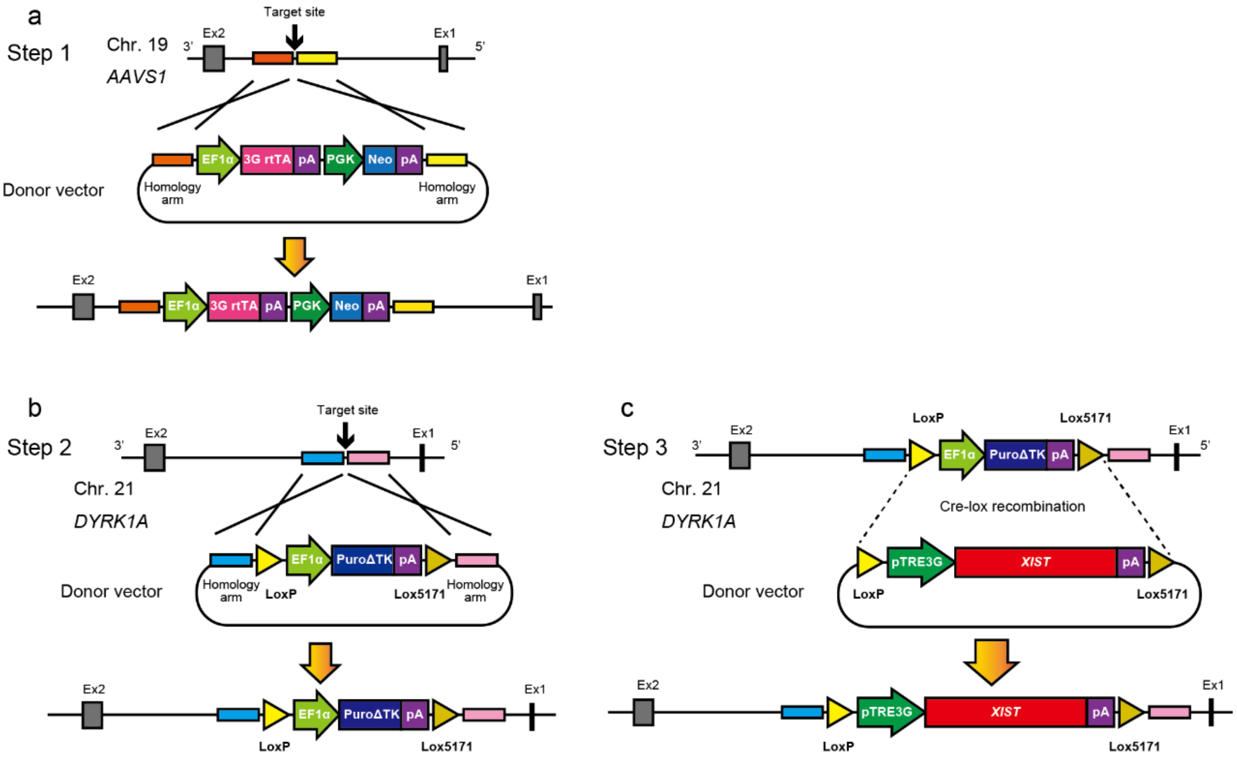

Supplementary Figure 1 | Generation of an *XIST*-mediated, chromosome 21-silencing system. a) (Step 1) Schematic representation depicting the targeted insertion of rtTA construct into the *AAVS1* locus of chromosome 19 in Tri21 iPSC line using a ZFN. Top: *AAVS1* gene locus including exons (grey boxes), ZFN-target sites (black arrow), and homology arms (orange and yellow bars). Middle: donor vector including homology arms, a human EF1α promoter (light-green arrow), the rtTA (pink box), poly(A) sequences (purple boxes), a human PGK promoter (green arrow), and the neomycin-resistance gene (Neo, blue box). Bottom: schematic of the rtTA-inserted *AAVS1* locus. **b)** (Step 2) Targeted insertion of the exchange cassette into the *DYRK1A* locus on chromosome 21. Top: *DYRK1A* gene locus including exons (grey boxes), CRISPR–Cas9-target sites (black arrow), and homology arms (blue and pink bars). Middle: donor vector including homology arms, the *loxP* sequence (yellow triangle), drug-resistance genes (PuroΔTK, blue box), poly(A) sequences, and the *lox5171* site (dark yellow triangle). Bottom: schematic of the exchange cassette-inserted *DYRK1A* locus. **c)** (Step 3) Cre recombinase-mediated cassette exchange used to insert human *XIST* cDNA. Top: Exchange cassette-inserted *DYRK1A* locus in chromosome 21. Middle: donor vector including the *loxP* sequence, the tetracycline-response element (green arrow, pTRE3G), human *XIST* cDNA (red box), poly(A) sequences, and the *lox5171* sequence. Bottom: schematic of the *XIST*-inserted *DYRK1A* locus.

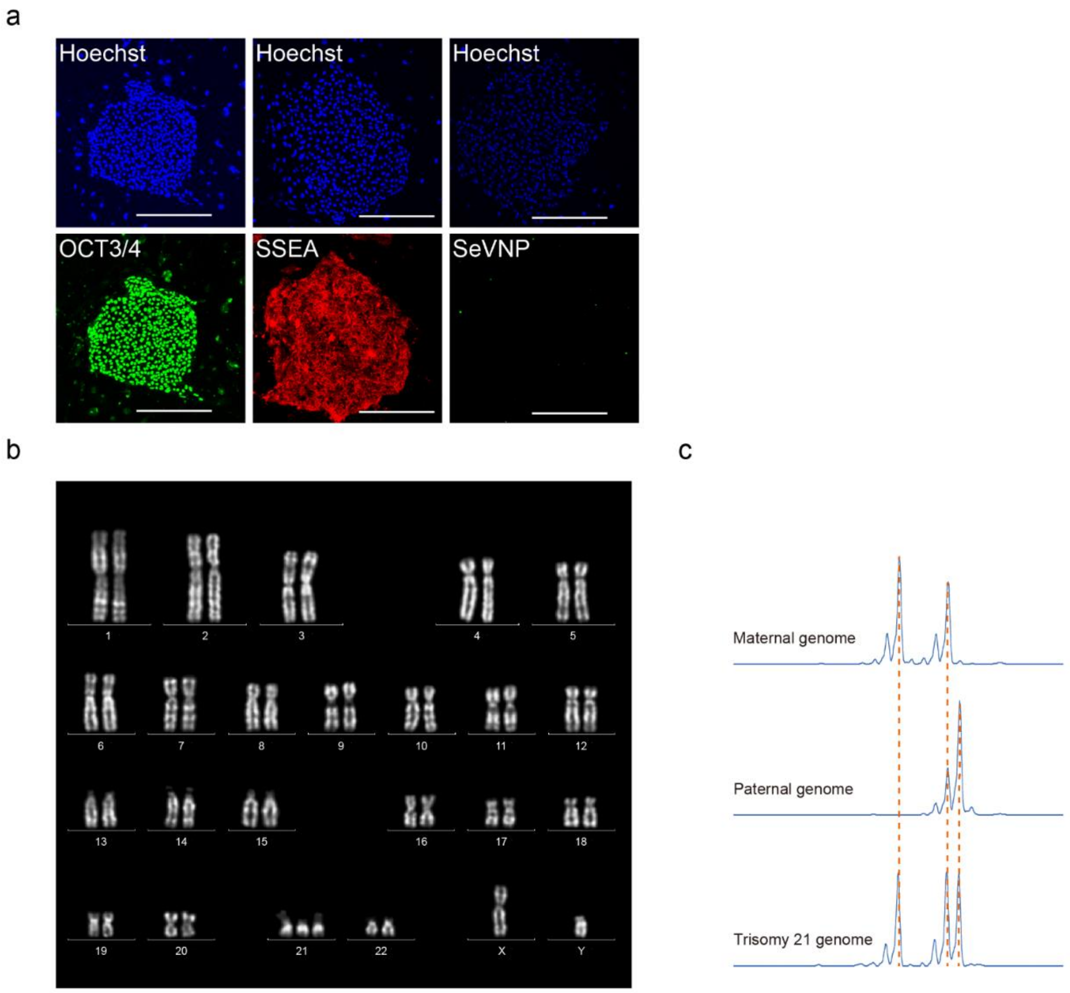

Supplementary Figure 2 | Analysis of an *XIST*-inserted trisomy 21 iPSC line. a) Immunocytochemical staining of the *XIST*-Tri21 iPSC line using specific antibodies against OCT3, OCT4, SSEA, and Sendai virus NP protein (SeVNP). Nuclei were stained with Hoechst 33342. Scale bars: 500 µm. **b)** Karyotype analysis of the *XIST*-Tri21 iPSC line. **c)** STR analysis of the *XIST*-Tri21 iPSC line. The STR sequence of the *RUNX1* locus in chromosome 21 was compared between a patient with DS and his parents' genomes.

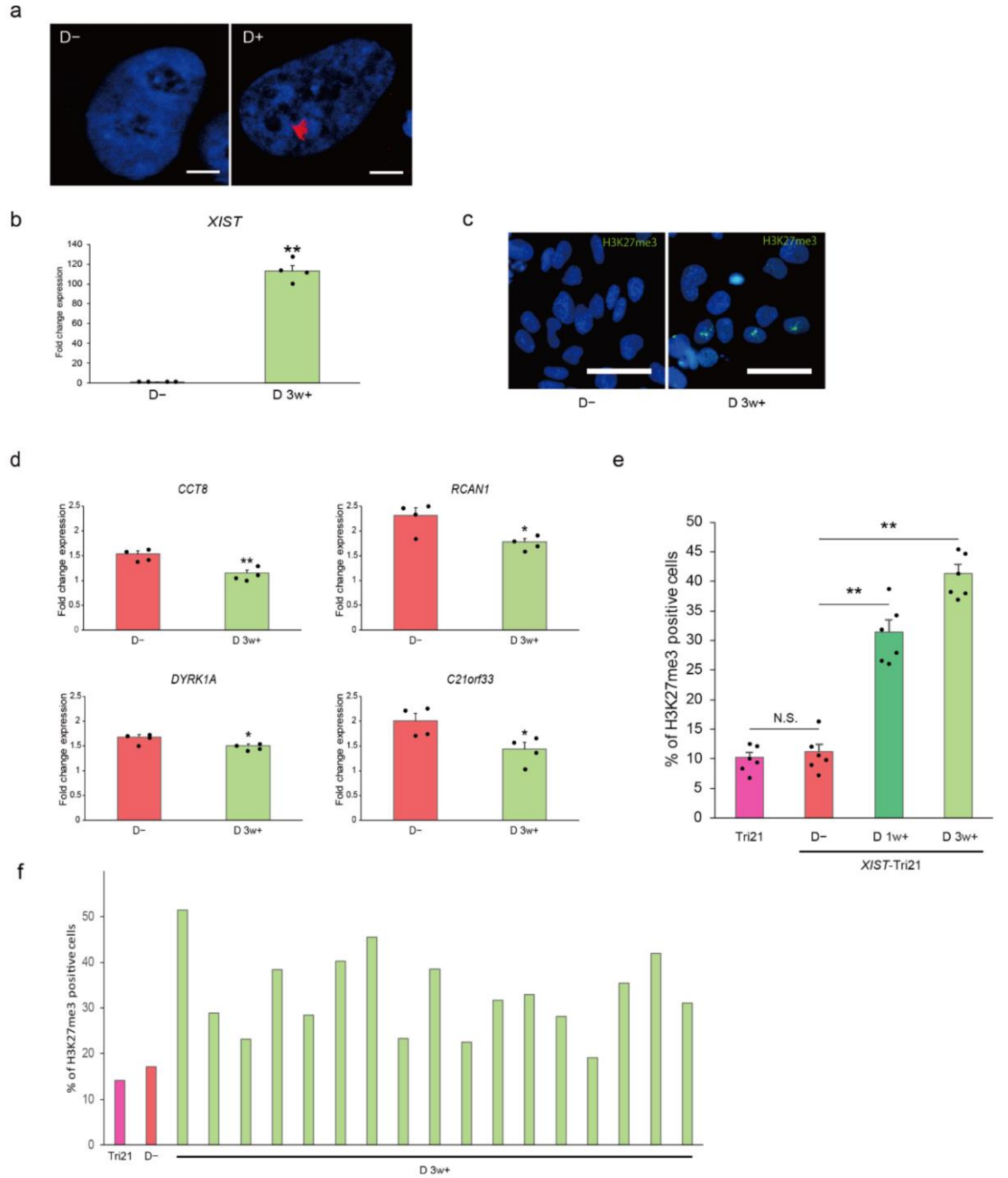

Supplementary Figure 3 | Dox treatment-induces chromosome silencing in the *XIST*-Tri21 iPSC line. a) FISH analysis of *XIST* RNA in untreated (D−) *XIST*-Tri21 iPSCs and *XIST*-Tri21 iPSCs treated with Dox (D+) for 3 weeks. *XIST* RNA was labelled with a Dylight594-conjugated probe (red). Nuclei were stained with DAPI. Scale bars: 5 µm. **b)** Relative expression levels of *XIST* in untreated (D−) and Dox-treated (D+) *XIST*-Tri21 iPSC lines (n = 4 experiments per cell line). Gene-expression levels were normalised to those of the D− cell lines. Error bars represent the SEM. **c)** Immunocytochemical staining of the *XIST*-Tri21 iPSC line using an anti-H3K27me3 antibody. Nuclei were stained with Hoechst 33342. Scale bars: 50 µm. **d)** Relative expression levels of genes on chromosome 21 in untreated (D−) and Dox-treated (D+) *XIST*-Tri21 iPSC lines (n = 4 experiments per line). Gene-expression levels were normalised to

those of cDi21 lines (n = 4 experiments per cell line). Error bars represent the SEM. **e)** Percentage of H3K27me3-positive cells in Tri21 iPSCs, untreated (D−) *XIST*-Tri21 iPSCs, and *XIST*-Tri21 iPSCs, treated with Dox for 1 week (D 1w+) or 3 weeks (D 3w+). Less than 45% of *XIST*-Tri21 iPSCs were H3K27me3-positive even after 3 weeks of Dox administration (n = 6 experiments per cell line). Two *XIST*-Tri21 lines were used for each experiment (**b, d**, **e**). **f)** Percentage of H3K27me3-positive Tri21 iPSCs, untreated (D−) *XIST*-Tri21 iPSCs, and (D 3w+) *XIST*-Tri21 iPSCs after single-cell cloning. A relatively low efficiency of H3K27me3 induction was continuously observed in single-cell-derived clones of *XIST*-Tri21 iPSCs. Error bars represent the SEM. The data shown were analysed using Student's t-test (**d**, **e**) or Welch's two-sample t-test (**b**). *$P < 0.05$, **$P < 0.01$; N.S., not significant ($P > 0.05$).

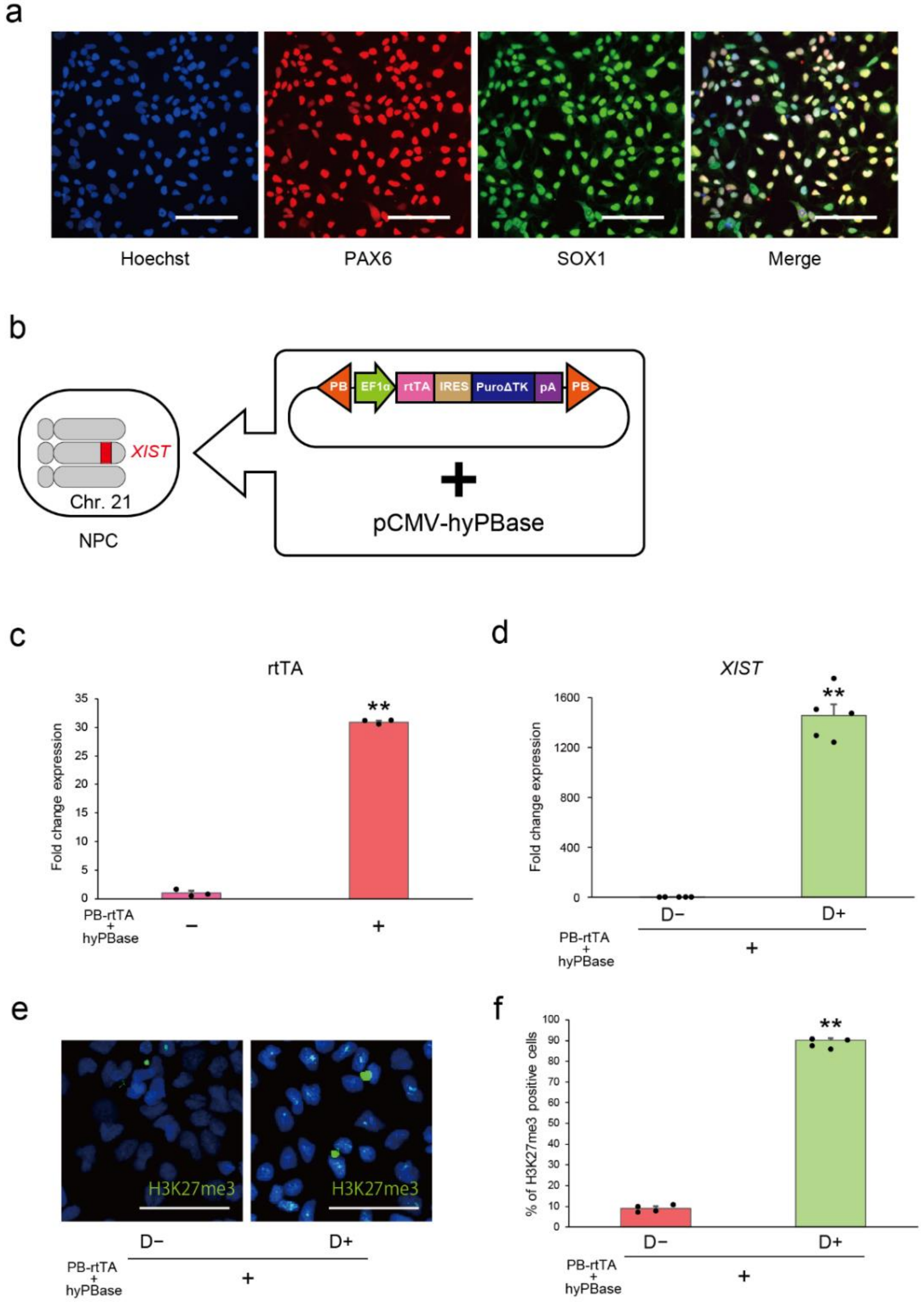
a
Hoechst
PAX6
SOX1
Merge
b
PB
EF1α
rtTA
IRES
PuroΔTK
pA
PB
XIST
Chr. 21
NPC
pCMV-hyPBase
c
rtTA
Fold change expression
**
PB-rtTA + hyPBase
−
+
d
XIST
Fold change expression
**
D−
D+
PB-rtTA + hyPBase
+
e
H3K27me3
H3K27me3
D−
D+
PB-rtTA + hyPBase
+
f
% of H3K27me3 positive cells
**
D−
D+
PB-rtTA + hyPBase
+

Supplementary Figure 4 | Additional transfection of rtTA into *XIST*-Tri21 NPCs. a) Immunocytochemical staining of *XIST*-Tri21 NPCs using PAX6- and SOX1-specific antibodies. Nuclei were stained with Hoechst 33342. Scale bars: 100 µm. **b)** Schematic depicting the additional transfection of an rtTA using a PB transposon vector and a hyperactive PB transposase into *XIST*-Tri21 NPCs. **c)** Relative rtTA-expression levels in *XIST*-Tri21 NPCs, with or without rtTA transfection (n = 3 experiments per cell line). rtTA expression from the gene cassette inserted into the *AAVS1* safe harbour locus was lost in NPCs, but restored by the transduction of PB-rtTA. Expression was normalised to that of untransfected cell lines. Error bars represent the SEM. **d)** Relative expression levels of *XIST* RNA in rtTA-transfected *XIST*-Tri21 NPCs. D+ cells were treated with Dox for 5 days. Expression levels were normalised to those of Dox-untreated lines (n = 5 experiments per cell line). **e)** Immunocytochemistry of rtTA-transfected *XIST*-Tri21 NPCs using an H3K27me3-specific antibody. D+ cells were treated with Dox for 5 days. Nuclei were stained with Hoechst 33342. Scale bars: 50 µm. **f)** Percentage of H3K27me3-positive cells in rtTA-transfected *XIST*-Tri21 NPCs. D+ cells were treated with Dox for 5 days (n = 4 experiments per cell line). Each of the data was obtained from three lines (**c, d, f**). Error bars represent the SEM. The data shown were analysed using Student's *t*-test (**c**, **f**) or Welch's two-sample *t*-test (**d**). $*P < 0.05$, $**P < 0.01$.

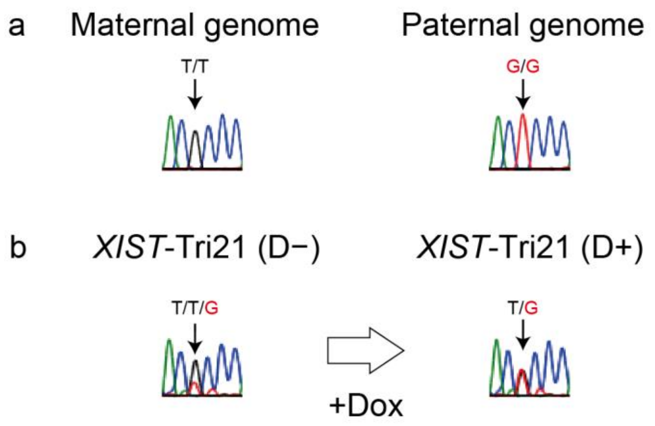

Supplementary Figure 5 | SNP analysis of mRNA extracted from *XIST*-Tri21 APCs, with or without Dox treatment, revealed the parental origin of the *XIST*-inserted chromosome 21. a) SNP analysis of the *ETS2* gene in chromosome 21 using the parents' genomic DNA showed T/T polymorphisms in the maternal genome and G/G polymorphisms in the paternal genome. **b)** SNP analysis of the *ETS2* gene using total cDNA (i.e., not genomic DNA) prepared from *XIST*-Tri21 APCs, with and without *XIST* expression. cDNA derived from *XIST*-Tri21 APCs without Dox treatment (D−) showed T/T/G polymorphisms, whereas cDNA derived from Dox-treated (chromosome-inactivated) *XIST*-Tri21 APCs lost a T allele, indicating that *XIST* cDNA was inserted into one of two maternal copies of chromosome 21 (hereafter the *XIST*-inserted maternal chromosome is referred to as M2).

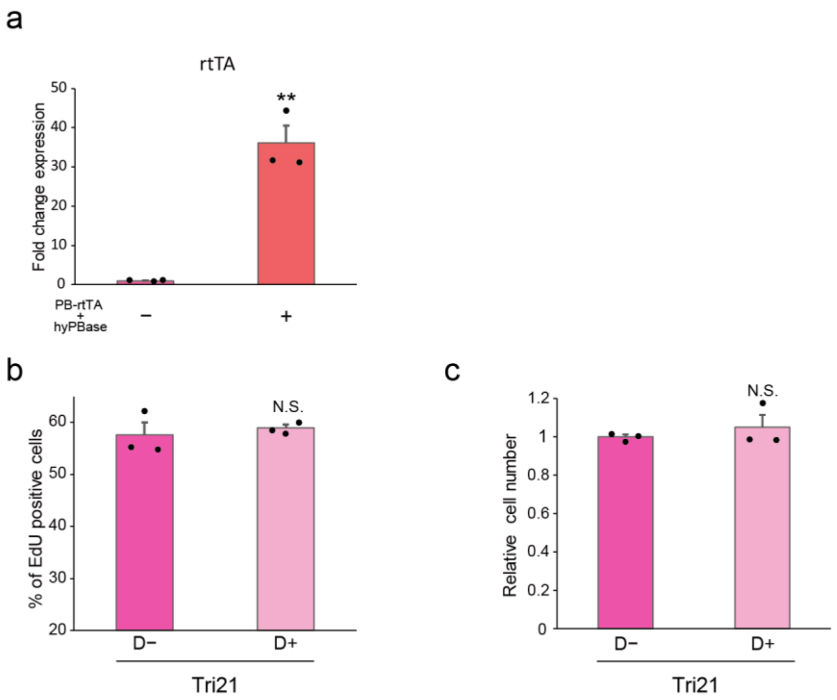

Supplementary Figure 6 | Dox administration does not effect the basal proliferative ability of Tri21 APCs. a) Relative rtTA-expression levels in *XIST*-Tri21 APCs. The expression levels were normalised to those of untransfected APCs (n = 3 experiments per cell line). Data were obtained from three lines. **b)** Percentage of EdU-positive Tri21 APCs (i.e., not containing the *XIST* transgene; n = 3 experiments per cell line). Dox was administered to the D+ line for 6 weeks. **c)** Relative numbers of Tri21 APCs 1 day after seeding. The cell numbers were normalised to those of the D− APC line (n = 3 experiments per cell line). Error bars represent the SEM. The data shown were analysed by Student's *t*-test (**b**, **c**) or Welch's two-sample *t*-test (**a**). *$P < 0.05$, **$P < 0.01$; N.S., not significant ($P > 0.05$).

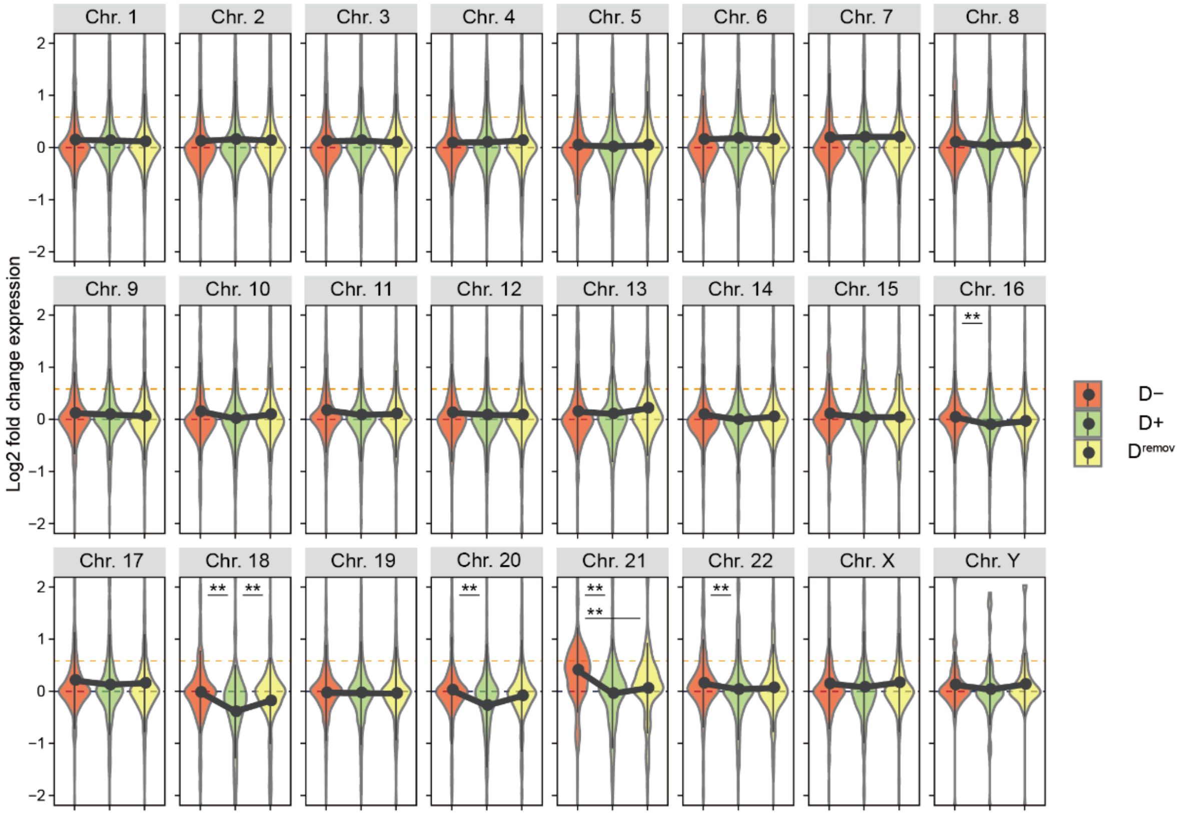

Supplementary Figure 7 | Transcriptional profiling of each chromosome in Dox-treated *XIST*-Tri21 APCs. Violin plots of relative log-transformed expression ratios for genes in each chromosome in *XIST*-Tri21 APC lines (n = 3 per cell line). D− cell (red); D+ cell (green); D^{remov} cell lines (yellow). Gene-expression levels with positive read counts (as determined by RNA-seq analysis) were normalised to those of cDi21 lines. The upper orange dashed lines indicate a ratio of 1.5, whereas the lower black dashed lines indicate a ratio of 1.0. The plots show mean expression levels with error bars indicating the SD. The data shown were analysed by the Kruskal–Wallis test with Bonferroni's correction. $**P < 0.01$

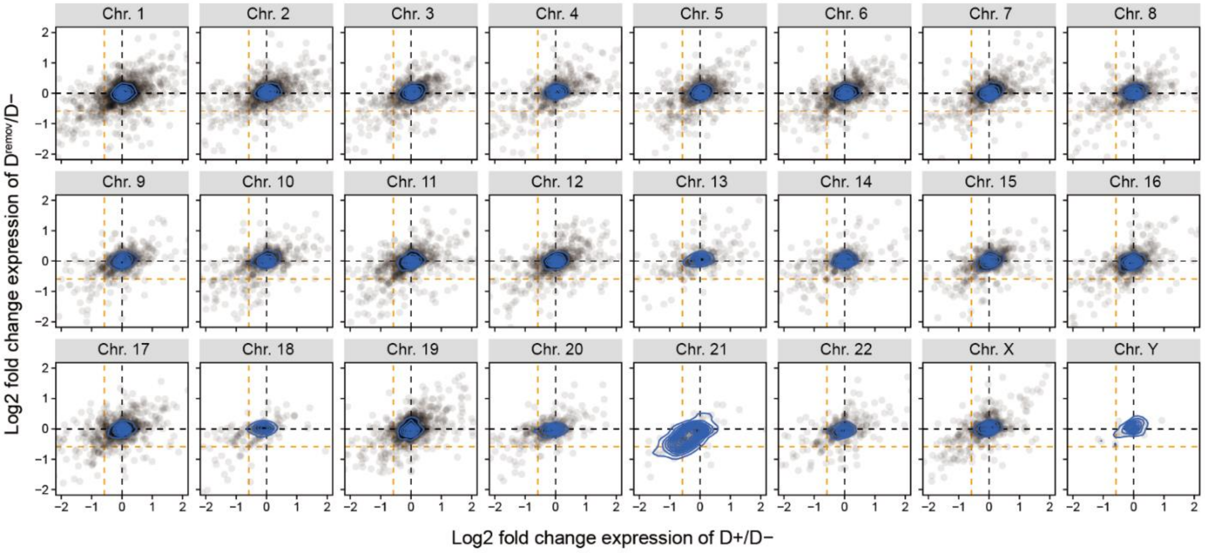

Supplementary Figure 8 | Transcriptional profiling showed sustained gene suppression in *XIST*-Tri21 APC lines after Dox removal. Log2-fold changes in the mean gene-expression ratios (Dox-treated lines: Dox-untreated lines, X-axis; D^{remov}: Dox-untreated lines, Y-axis). The grey data points represent the full set of 18,440 genes with positive read, as determined by RNA-seq, with the point densities for all sets represented by blue line contours. The orange dashed lines indicate a two-thirds decrease in the gene-expression levels for each cell line.

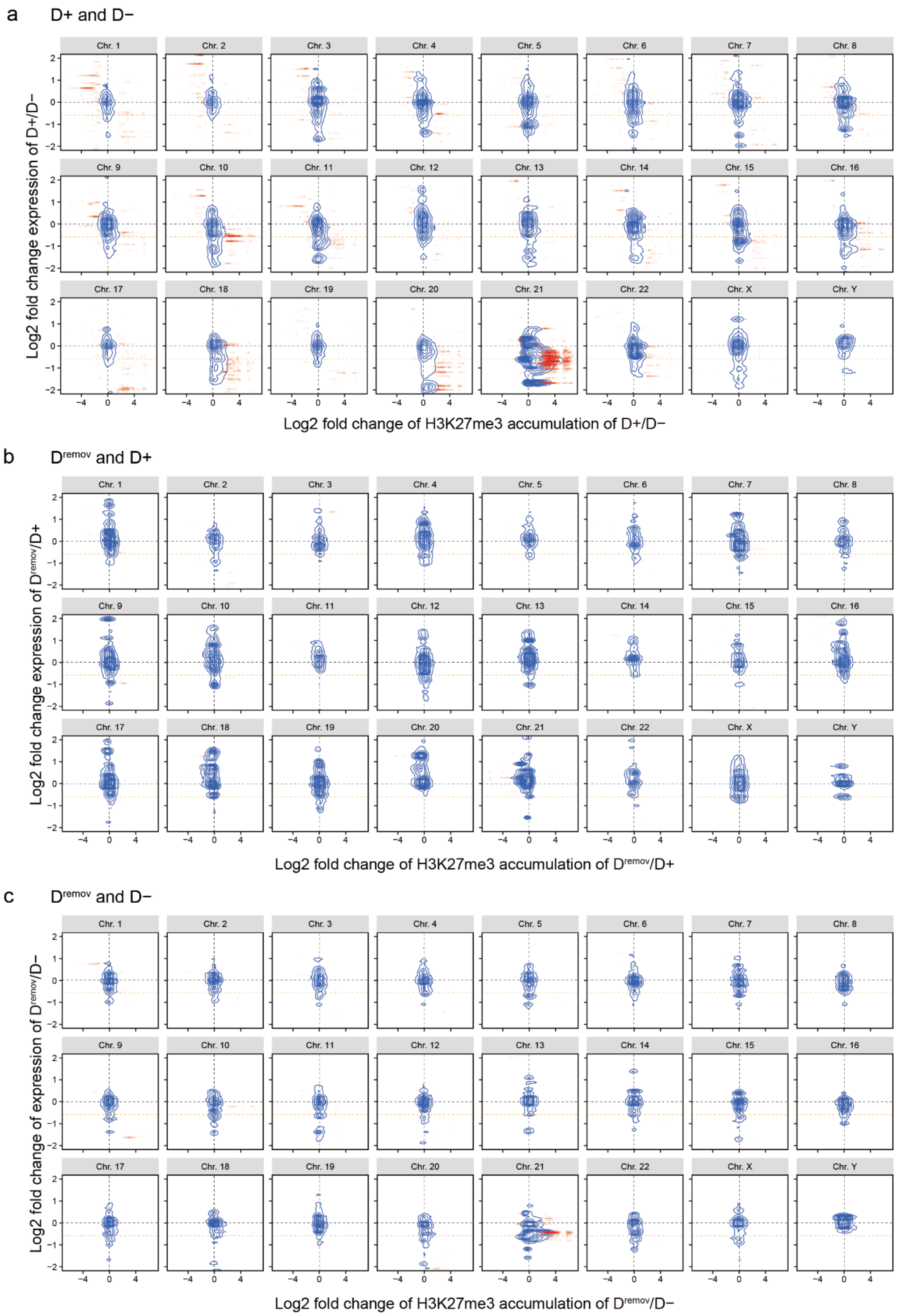
a
D+ and D−
Chr. 1
Chr. 2
Chr. 3
Chr. 4
Chr. 5
Chr. 6
Chr. 7
Chr. 8
Chr. 9
Chr. 10
Chr. 11
Chr. 12
Chr. 13
Chr. 14
Chr. 15
Chr. 16
Chr. 17
Chr. 18
Chr. 19
Chr. 20
Chr. 21
Chr. 22
Chr. X
Chr. Y
Log2 fold change expression of D+/D−
Log2 fold change of H3K27me3 accumulation of D+/D−
b
D^{remov} and D+
Log2 fold change expression of D^{remov}/D+
Log2 fold change of H3K27me3 accumulation of D^{remov}/D+
c
D^{remov} and D−
Log2 fold change of expression of D^{remov}/D−
Log2 fold change of H3K27me3 accumulation of D^{remov}/D−

Supplementary Figure 9 | Integrative analysis of the RNA-seq and ChIP-seq data revealed a negative correlation between H3K27me3 enrichment and transcriptional silencing in D+ and D^{remov} *XIST*-Tri21 APC lines. a–c) H3K27me3 accumulation within each gene body and 2 kb upstream of the transcription start site were compared between D+ and D− cell lines (**a**), D^{remov} and D+ cell lines (**b**), and D^{remov} and D− *XIST*-Tri21 APC lines (**c**). Genes with a significant difference in H3K27me3 accumulation between two lines are represented with red data points, with the density for all pair points shown by blue line contours. The orange dashed lines indicate a two-thirds decrease in the gene-expression level of each cell line. With the ChIP-seq data, the FDRs were calculated using the Benjamini–Hochberg method, and FDR < 0.05 was considered to reflect a statistically significant difference.

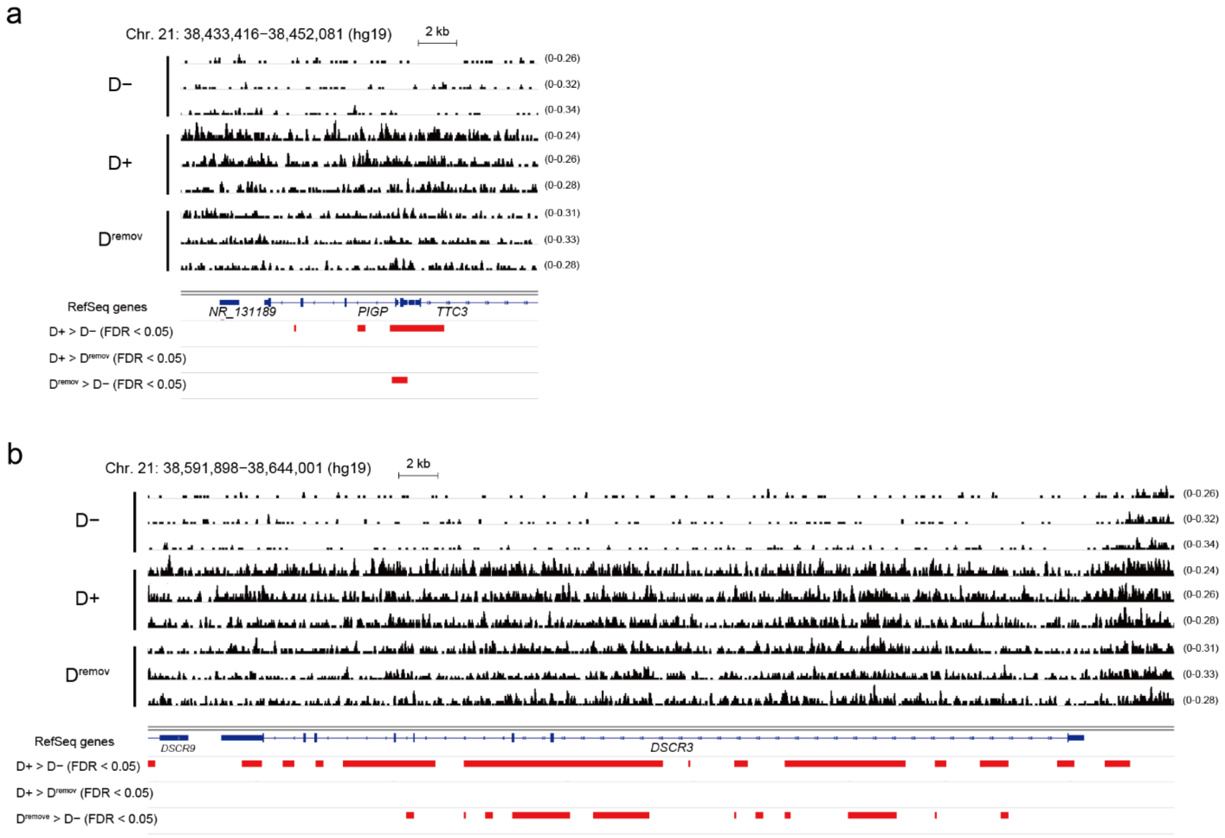

Supplementary Figure 10 | Map showing the distribution of H3K27me3 modifications in *PIGP* and *DSCR3* in *XIST*-Tri21 APCs. a, b) Integrative Genomics Viewer screenshot of H3K27me3 ChIP-seq track peaks for *PIGP* (**a**) and *DSCR3* (hg19) (**b**) in Dox-untreated cell lines, Dox-treated cell lines, and D^{remov} cell lines. The Y-axis shows the number of fragments per base pair per million reads. (Red bars) The distribution of the regions with significantly higher H3K27me3 accumulation in each comparison. The FDRs were calculated using the Benjamini–Hochberg method. FDR < 0.05 was considered to reflect a statistically significant difference.

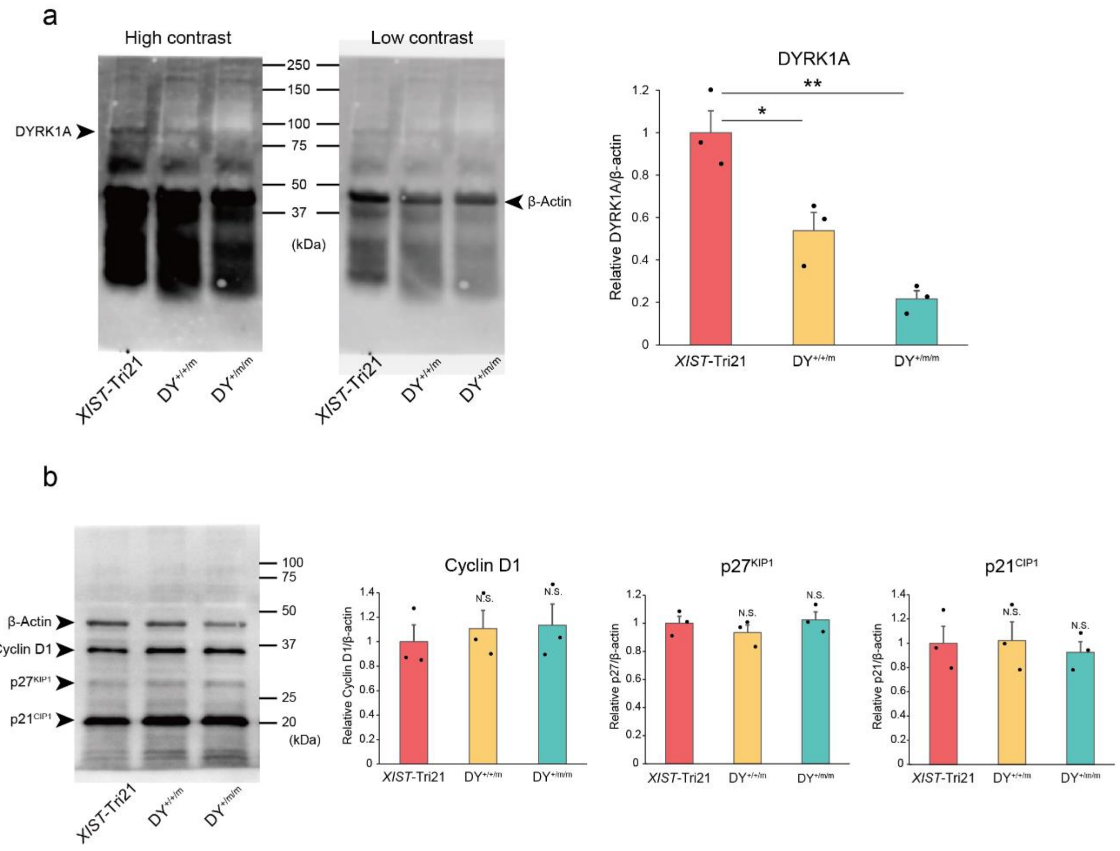

Supplementary Figure 11 | Targeted deletion of *DYRK1A* in APCs had little effect on cell cycle-related protein levels. a, b) Immunoblot analysis of DYRK1A (**a**) and cell cycle-related proteins (Cyclin D1, p27^{KIP1}, p21^{CIP1}) (**b**) in *DYRK1A*-targeted *XIST*-Tri21 APCs. The high-contrast and low-contrast images were taken from the same membrane. β-Actin was used as a loading control and expression levels were normalised to that of the *XIST*-Tri21 line (n = 3 experiments per cell line). Each of the data was obtained from three lines. Error bars represent the SEM. Data were analysed by Student's *t*-test. *$P < 0.05$, **$P < 0.01$; N.S., not significant ($P > 0.05$).

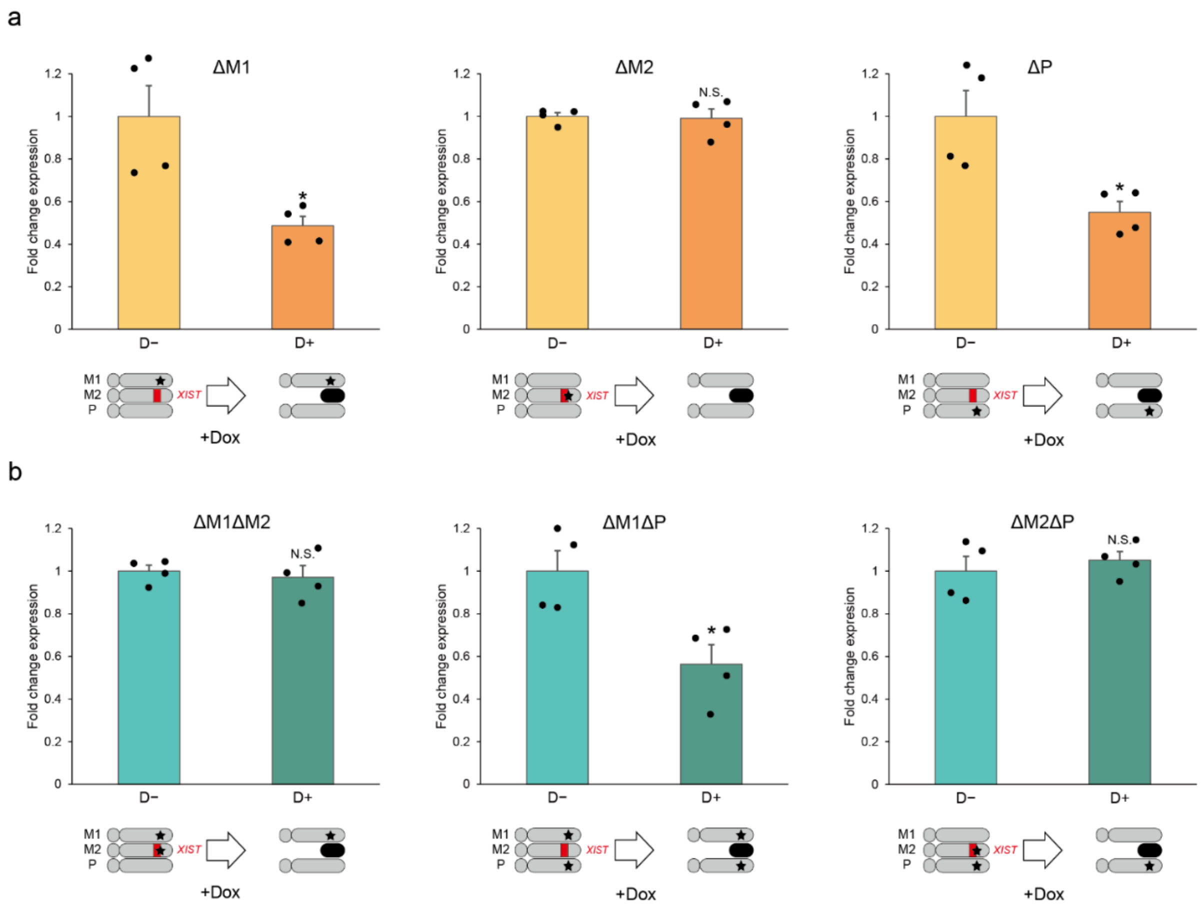

Supplementary Figure 12 | *DYRK1A* expression levels in DY$^{+/+/m}$- and DY$^{+/m/m}$- *XIST*-Tri21 APCs depend on the copy numbers of active *DYRK1A* genes. a, b) Relative *DYRK1A*-expression levels in DY$^{+/+/m}$- (**a**) and DY$^{+/m/m}$- (**b**) *XIST*-Tri21 APCs, with or without Dox treatment. Expression levels were normalised to that of Dox-untreated lines (n = 4 experiments per cell line). Error bars represent the SEM. The data shown were analysed by Student's *t*-test. *$P < 0.05$; N.S., not significant ($P > 0.05$). (Lower) Schematic depiction of the combination of targeted *DYRK1A* alleles and inactivated chromosome 21. The parental origin of chromosome 21 is indicated (M1, M2, or P). Red rectangle, *XIST* transgene; black stars, *DYRK1A*-target sites; black chromosomes, inactivated chromosome 21. Expected copy numbers of the active *DYRK1A* gene decreased from two to one in ΔM1- and ΔP-DY$^{+/+/m}$ APC lines, and from one to zero in ΔM1ΔP-DY$^{+/m/m}$ APC lines after Dox treatment. In contrast, the expected copy numbers of the active *DYRK1A* gene remained unchanged as two in the ΔM2-DY$^{+/+/m}$ APC line, or as one in the ΔM1ΔM2- and ΔM2ΔP-DY$^{+/m/m}$ APC lines.

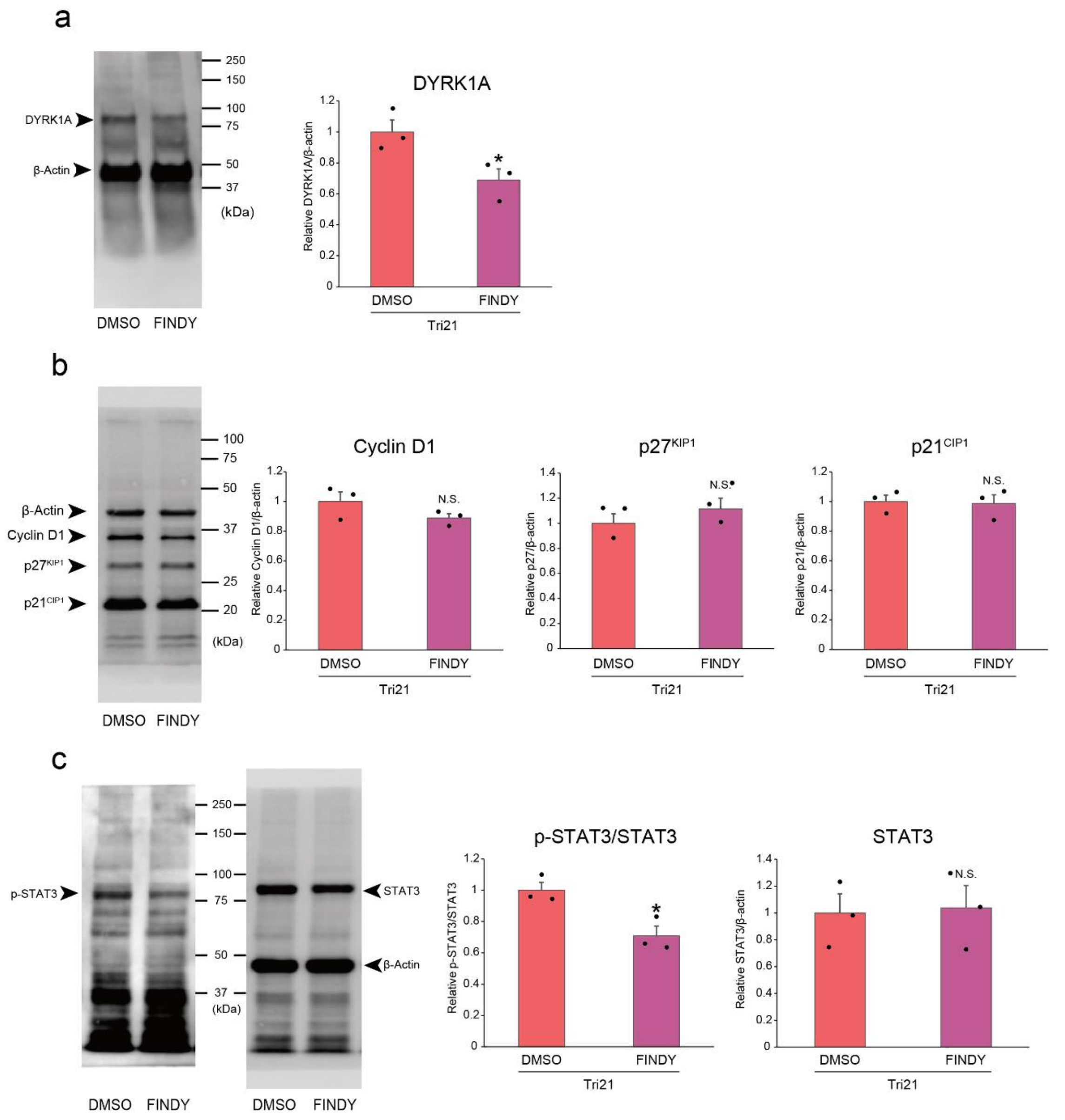

Supplementary Figure 13 | DYRK1A inhibitor decreases p-STAT3 levels in Tri21 APCs. a–c) Immunoblot analysis of DYRK1A (**a**), cell cycle-related proteins (Cyclin D1, $p27^{KIP1}$, $p21^{CIP1}$) (**b**), and p-STAT3 and STAT3 (**c**) in Tri21 APCs after 2-day treatment with FINDY (2.5 μM). (**c**) The left blotted membrane was probed with an anti-p-STAT3 antibody. The right blotted membrane was stripped and re-probed with anti-STAT3 and anti-β-actin antibodies. β-Actin was used as a loading control and expression levels were normalised to that of Tri21 APCs without FINDY treatment (n = 3 experiments per condition). Error bars represent the SEM. Data were analysed by Student's *t*-test. *$P < 0.05$; N.S., not significant ($P > 0.05$).

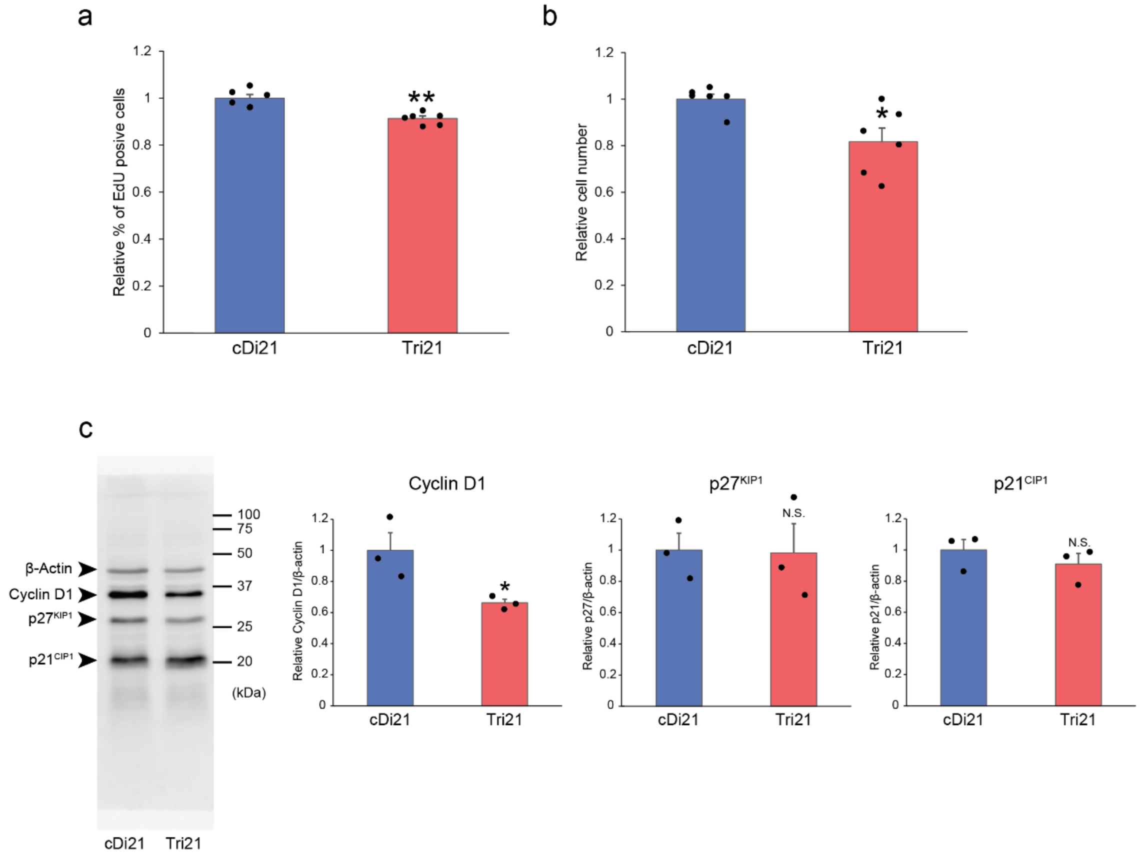

Supplementary Figure 14 | Tri21 NPCs exhibit impaired proliferation and decreased levels of Cyclin D1. a) Percentage of EdU-positive NPCs, which was normalised to that of the cDi21 cell lines (n = 6 experiments per cell line). **b)** Relative numbers of NPCs 1 day after seeding. Cell numbers were normalised to that of the cDi21 cell lines (n = 6 experiments per cell line). **c)** Immunoblot analysis of Cyclin D1, $p27^{KIP1}$ and $p21^{CIP1}$ in NPCs. β-Actin was used as a loading control and expression levels were normalised to that of the cDi21 cell line (n = 3 experiments per cell line). Error bars represent the SEM. Data were analysed by Student's *t*-test (**a**, **c**) or Welch's two-sample *t*-test (**b**). $*P < 0.05$, $**P < 0.01$; N.S., not significant ($P > 0.05$).

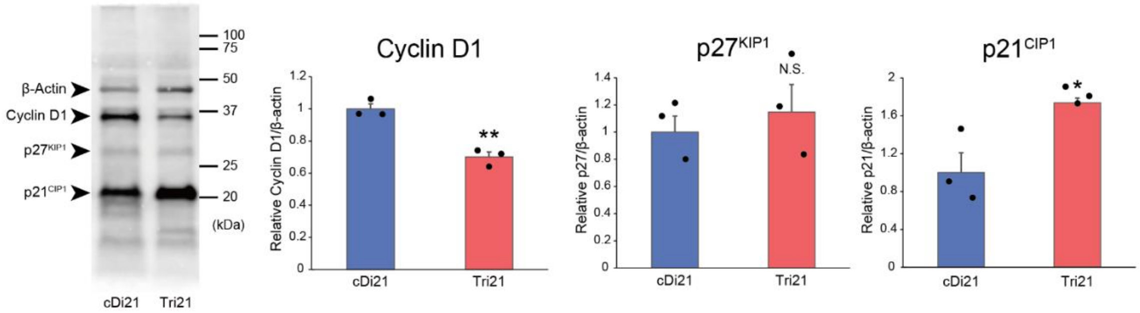

Supplementary Figure 15 | Changes in Cyclin D1 and p21 levels in Tri21 APCs. Immunoblot analysis of cell cycle-related proteins (Cyclin D1, $p27^{KIP1}$, $p21^{CIP1}$) in APCs. β-Actin was used as a loading control and expression levels were normalised to that of the cDi21 cell line (n = 3 experiments per cell line). Each of the data was obtained from two lines. Error bars represent the SEM. Data were analysed by Student's *t*-test. *$P < 0.05$, **$P < 0.01$; N.S., not significant ($P > 0.05$).

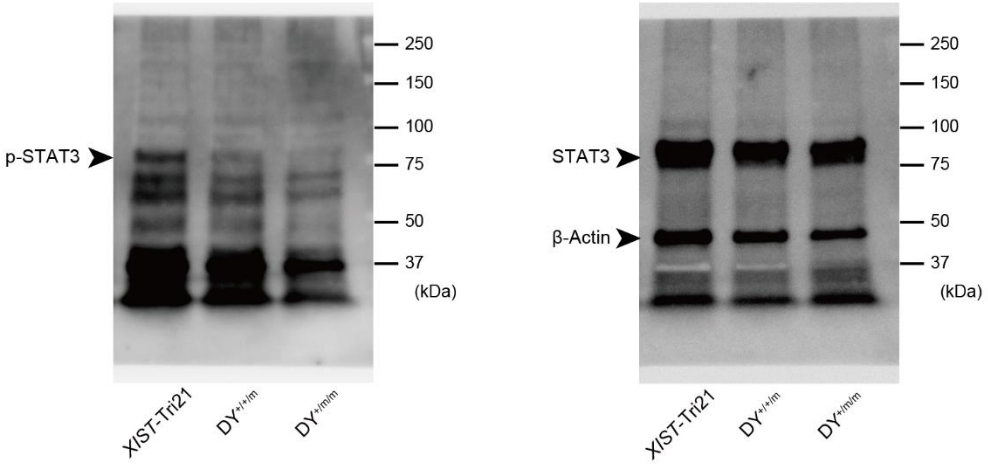

Supplementary Figure 16 | Full immunoblots related to the data shown in Fig. 5g. Full immunoblot images for p-STAT3 and STAT3. β-Actin was detected as a loading control. The left blotted membrane was probed with an anti-p-STAT3 antibody. The right blotted membrane was stripped and re-probed with anti-STAT3 and anti-β-actin antibodies.

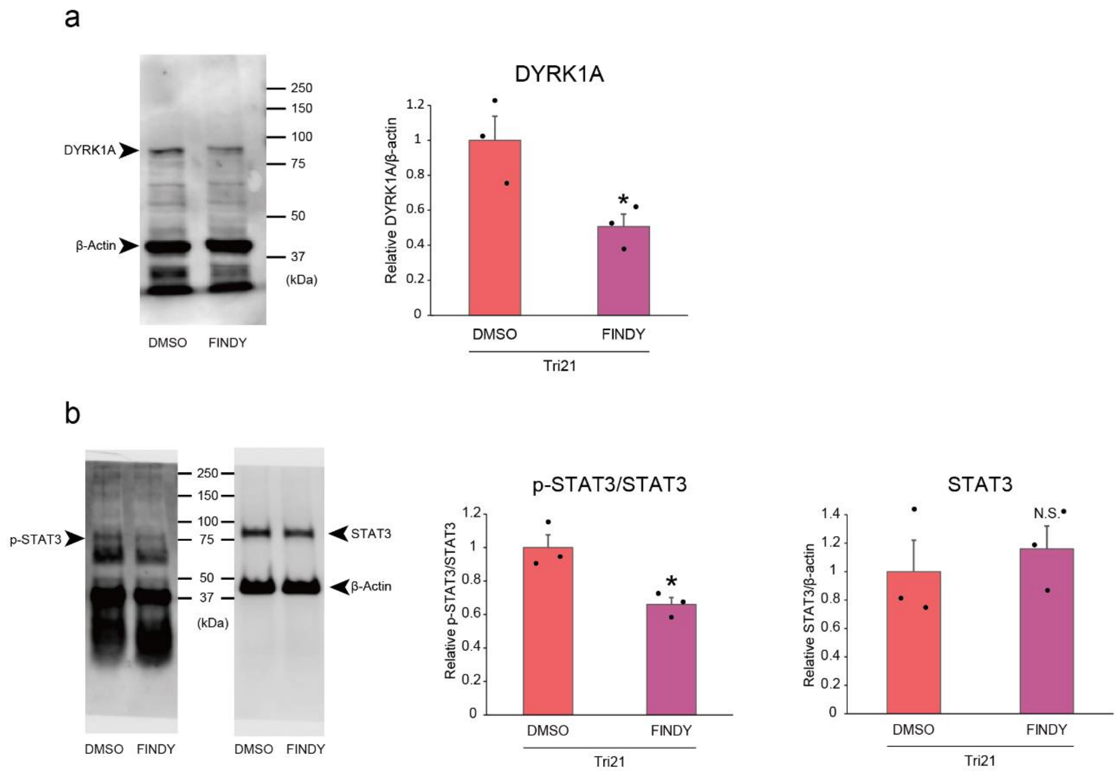

Supplementary Figure 17 | DYRK1A inhibitor decreases p-STAT3 levels in Tri21 NPCs. a, b) Immunoblot analysis of DYRK1A (**a**) and p-STAT3 and STAT3 (**b**) in Tri21 NPCs after 2-day treatment with FINDY (2.5 μM). (**b**) The left blotted membrane was probed with an anti-p-STAT3 antibody. The right blotted membrane was stripped and re-probed with anti-STAT3 and anti-β-actin antibodies. β-Actin was used as a loading control and expression levels were normalised to that of Tri21 NPCs without FINDY treatment (n = 3 experiments per condition). Error bars represent the SEM. Data were analysed by Student's *t*-test. *$P < 0.05$; N.S., not significant ($P > 0.05$).

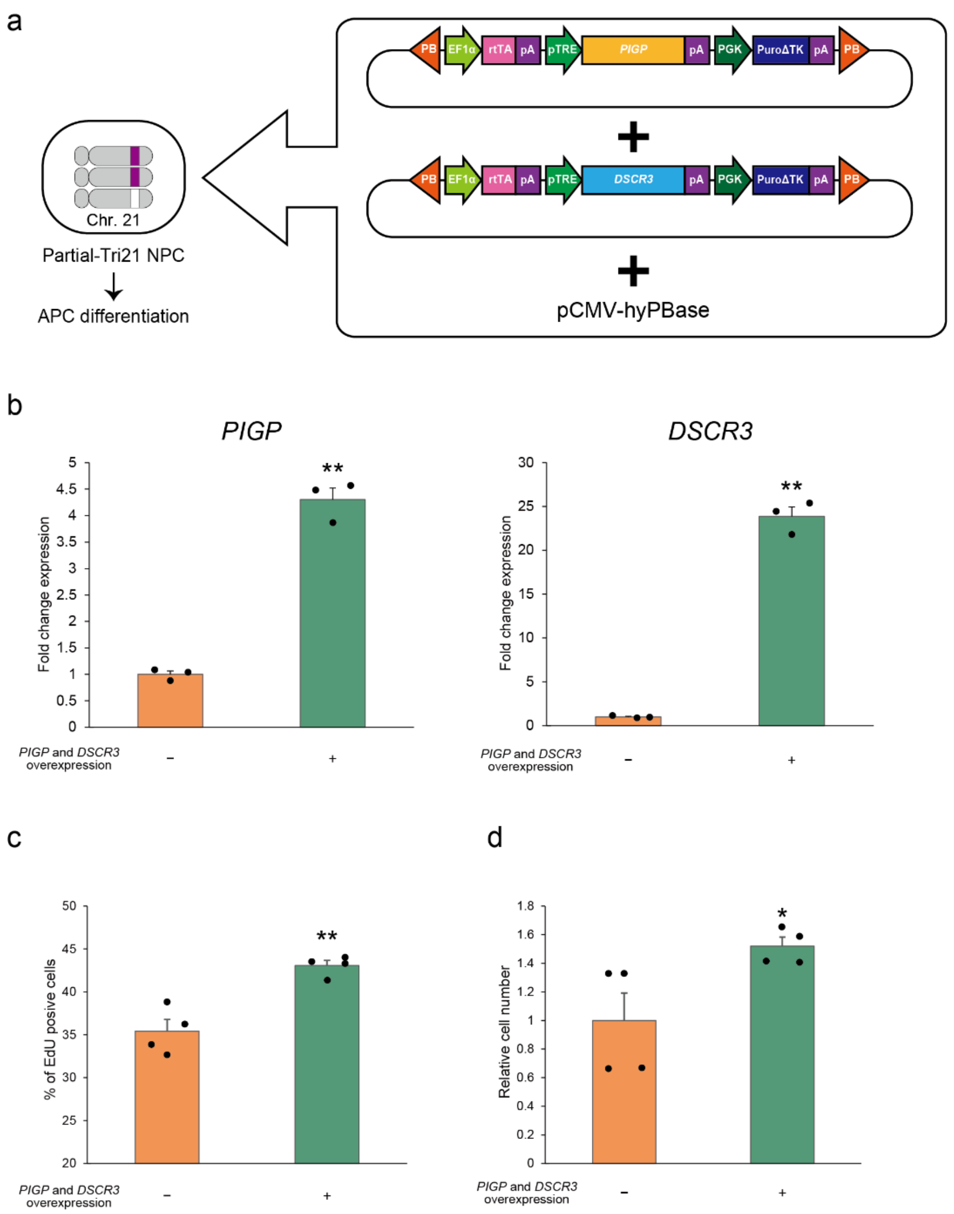

Supplementary Figure 18 | Co-overexpression of *PIGP* and *DSCR3* promotes Tri21 APC proliferation. a) Schematic depicting the transfection of Dox-inducible *PIGP* and *DSCR3* transgenes using PB transposon vectors and a hyperactive PB transposase into Partial-Tri21 NPCs. Transduced NPCs were differentiated into APCs. **b)** Partial-Tri21 APCs stably expressing the *PIGP* and *DSCR3*-overexpression vectors. *PIGP* and *DSCR3* overexpression was induced by a 6-week Dox treatment. Expression levels were normalised to that of untreated APCs (n = 3 experiments per condition). **c, d)** Percentage of EdU-positive cells (**c**) and relative cell numbers 1 day after seeding (**d**) in *PIGP* and *DSCR3*-overexpressing Partial-Tri21 APCs (n = 4 experiments per condition). Cell numbers were normalised to that of untreated APCs. Error bars represent the SEM. Data were analysed by Student's *t*-test (**c**, **d**) or Welch's two-sample *t*-test (**b**). *$P < 0.05$, **$P < 0.01$.

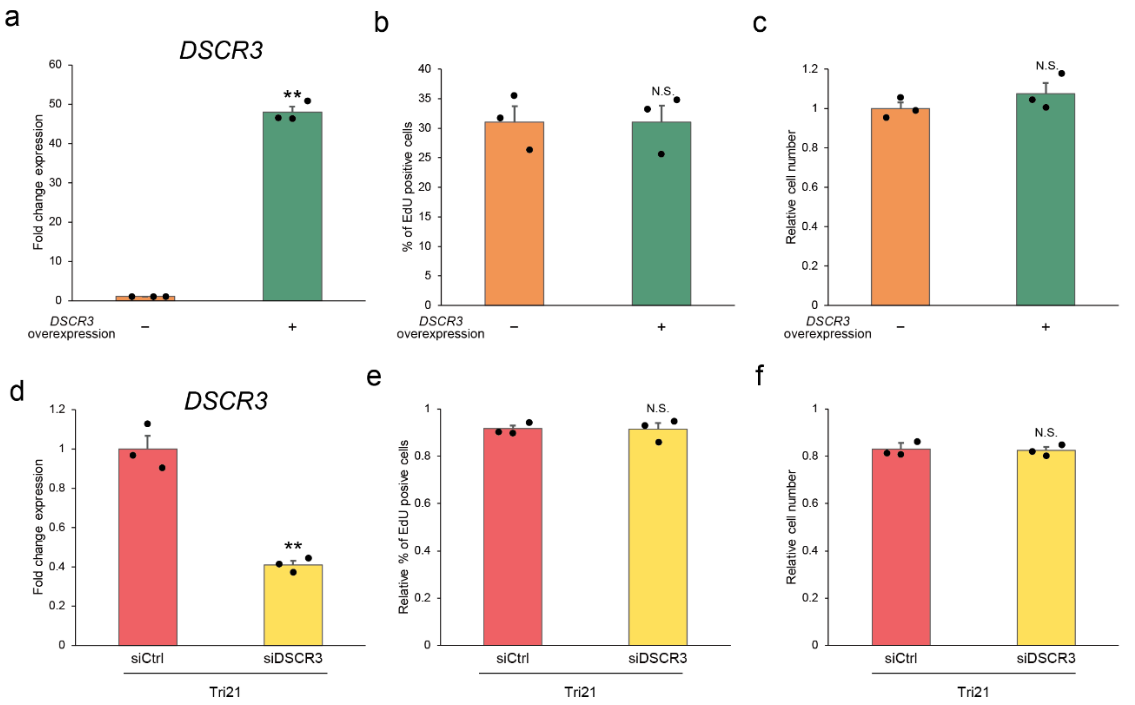

Supplementary Figure 19 | Neither overexpression nor knockdown of *DSCR3* affected Tri21 APC proliferation. A Dox-inducible *DSCR3* transgene was transduced into Partial-Tri21 NPCs using a PB transposon vector and a hyperactive PB transposase. Transfected NPCs were differentiated into APCs, and *DSCR3* overexpression was induced by a 6-week treatment with Dox. **a)** Relative *DSCR3*-expression levels in overexpression vector-transfected Partial-Tri21 APCs. The expression levels were normalised to those of untreated APCs (n = 3 experiments per condition). **b, c)** Percentage of EdU-positive cells (**b**) and relative cell numbers 1 day after seeding (**c**) in *DSCR3*-overexpressing Partial-Tri21 APCs (n = 3 experiments per condition). The cell numbers were normalised to those of untreated APC lines. **d)** Relative *DSCR3*-expression levels in siRNA-treated Tri21 APCs. The expression levels were normalised to those in control siRNA (siCtrl)-treated Tri21-APCs (n = 3 experiments per condition). **e, f)** Percentage of EdU-positive cells (**e**) and relative cell numbers 1 day after seeding (**f**) in siRNA-treated Tri21 APCs (n = 3 experiments per condition). The percentage of EdU-positive cells and cell numbers were normalised to that of the Tri21 cell line without siRNA treatment. siDSCR3, *DSCR3* siRNA. Error bars represent the SEM. The data shown were analysed by Student's t-test (**b**–**f**) or Welch's two-sample t-test (**a**). **P* < 0.05, ***P* < 0.01; N.S., not significant (*P* > 0.05).

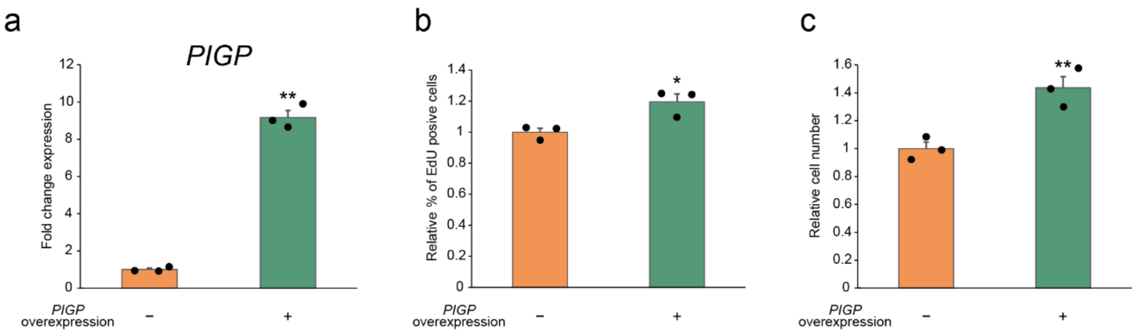

Supplementary Figure 20 | *PIGP* overexpression promotes Tri21 NPC proliferation. a) Partial-Tri21 NPCs stably expressing the *PIGP*-overexpression vector. *PIGP* overexpression was induced by a 5-day Dox treatment. Expression levels were normalised to that of untreated NPCs (n = 3 experiments per condition). **b, c)** Percentage of relative EdU-positive cells (**b**) and relative cell numbers 1 day after seeding (**c**) in *PIGP*-overexpressing Partial-Tri21 NPCs (n = 3 experiments per condition). The data were normalised to that of untreated NPCs. Error bars represent the SEM and the data were analysed by Student's *t*-test. *$P < 0.05$, **$P < 0.01$.

Supplementary Table 1 | Details of isogenic cell lines generated in this study (related to Figure 1).

Cell	Line	The number of line	The number of passage
	Tri21	1 line	
	XIST-Tri21	2 lines	
	cDi21 (M1 + P)	2 lines	
iPSC	cDi21 (M2 + P)	1 line	40 – 50
	Partial-Tri21	1 line	
	DY$^{+/+/m}$-*XIST*-Tri21	Each 1 line	
	DY$^{+/m/m}$-*XIST*-Tri21	Each 1 line	
	Tri21	1 line	
	XIST-Tri21	3 lines	
	cDi21 (M1 + P)	2 lines	
	cDi21 (M2 + P)	1 line	
NPC, APC	Partial-Tri21	3 lines	7 – 10 (NPC)
	DY$^{+/+/m}$-*XIST*-Tri21	Each 1 line	7 – 8 (APC)
	DY$^{+/m/m}$-*XIST*-Tri21	Each 1 line	
	PIGP overexpression	1 line	
	DSCR3 overexpression	1 line	
	PIGP and *DSCR3* co-overexpression	1 line	

Tri21 iPSCs were derived from a male patient with DS, from which two *XIST*-Tri21 iPSC lines were generated. *XIST*-Tri21 NPCs were independently transfected using a *piggyBac* vector encoding rtTA to generate three lines. cDi21 iPSC lines consisted of three lines, of which two had a deleted maternal allele 2 of chromosome 21 (cDi21 M1 + P) and one had a deleted maternal allele 1 (cDi21 M2 + P). Three Partial-Tri21 NPC lines were separately differentiated with a line of Partial-Tri21 iPSCs. The passage numbers for each cell line are indicated in the table.

Supplementary Table 2 | *XIST*-mediated silencing does not affect astrocyte and APC markers.

Gene	D−	D+	D^{remov}
GFAP	96.3	96.8	96.5
S100β	98.7	98.5	99.2
CD44	99.8	99.9	99.8
Vimentin	100.0	99.9	100.0

(%)

The average percentage of cells positive for GFAP, S100β, CD44, or vimentin in *XIST*-Tri21 APCs (n = 3 experiments per cell line). Each of the data was obtained from three lines. APCs of passage 8 were used for the analysis.

Supplementary Table 3 | PCA scores for genes on chromosome 21, based on the RNA-seq data.

Gene	Component 1	Component 2	Gene	Component 1	Component 2	Gene	Component 1	Component 2
SOD1	0.1272	0.0017	*CHAF1B*	0.0862	0.0504	*JAM2*	0.0428	0.1008
ITSN1	0.1253	0.0012	*WDR4*	0.0859	0.0244	*SLC19A1*	0.0427	0.0312
C21orf59	0.1231	0.0006	*PTTG1IP*	0.0857	0.0238	*MX1*	0.0417	0.1359
DONSON	0.1230	0.0049	*PRMT2*	0.0856	0.0513	*MCM3AP-AS1*	0.0414	0.0940
RRP1	0.1226	0.0032	*GART*	0.0846	0.1267	*LINC00515*	0.0393	0.0696
C21orf33	0.1201	0.0587	*YBEY*	0.0846	0.1090	*KCNJ6*	0.0393	0.0696
DYRK1A	0.1195	0.0035	*C21orf91*	0.0843	0.1172	*LCA5L*	0.0389	0.0121
DSCR3	0.1193	0.0265	*TRAPPC10*	0.0837	0.0108	*C21orf62*	0.0389	0.0096
HLCS	0.1188	0.0544	*HSF2BP*	0.0829	0.0029	*NRIP1*	0.0379	0.0499
PFKL	0.1163	0.0318	*RSPH1*	0.0825	0.0604	*B3GALT5*	0.0375	0.0926
N6AMT1	0.1160	0.0150	*UBE2G2*	0.0817	0.0247	*LINC00162*	0.0373	0.0287
U2AF1	0.1147	0.0135	*COL18A1*	0.0813	0.1031	*GRIK1-AS2*	0.0370	0.1087
BACH1	0.1144	0.0168	*SH3BGR*	0.0804	0.1101	*ADARB1*	0.0365	0.1455
MRPL39	0.1139	0.0499	*WRB*	0.0801	0.0691	*RCAN1*	0.0356	0.0998
ATP5O	0.1132	0.0480	*LSS*	0.0799	0.0712	*LOC101927797*	0.0354	0.1458
AGPAT3	0.1131	0.0146	*C21orf58*	0.0795	0.0741	*LRRC3-AS1*	0.0342	0.1065
CCT8	0.1126	0.0374	*PCNT*	0.0794	0.0772	*RIPK4*	0.0334	0.0893
URB1	0.1122	0.0309	*SON*	0.0793	0.1108	*LINC00316*	0.0319	0.0095
SCAF4	0.1120	0.0220	*MX2*	0.0789	0.0949	*LRRC3*	0.0314	0.1211
TMEM50B	0.1119	0.0427	*IFNGR2*	0.0771	0.0950	*ERG*	0.0298	0.0796
APP	0.1114	0.0522	*DSCAM*	0.0771	0.0267	*SIK1*	0.0294	0.0577
ATP5J	0.1110	0.0701	*BACE2*	0.0771	0.0198	*ETS2*	0.0285	0.0725
DNAJC28	0.1104	0.0351	*PSMG1*	0.0770	0.1219	*GRIK1*	0.0285	0.0725
CHODL	0.1080	0.0576	*CBR3-AS1*	0.0747	0.1290	*RIPPLY3*	0.0285	0.0725
MORC3	0.1067	0.0534	*FAM207A*	0.0729	0.0232	*RUNX1-IT1*	0.0235	0.1391
LOC642852	0.1059	0.0321	*SLC37A1*	0.0711	0.0480	*PDE9A*	0.0218	0.1087
PDXK	0.1058	0.0450	*EVA1C*	0.0701	0.0695	*NCAM2*	0.0211	0.0077
C21orf2	0.1058	0.0577	*TFF2*	0.0697	0.0982	*LOC100129027*	0.0209	0.1630
BTG3	0.1051	0.0127	*AIRE*	0.0693	0.0185	*LOC100133286*	0.0193	0.1345
PWP2	0.1046	0.0069	*LINC00189*	0.0693	0.0185	*KCNE2*	0.0189	0.1329
PIGP	0.1044	0.0314	*TMPRSS15*	0.0688	0.0761	*DSCR9*	0.0187	0.0942
SPATC1L	0.1041	0.0367	*LOC100506403*	0.0687	0.0112	*LINC00158*	0.0185	0.0702
USP16	0.1033	0.0508	*LINC00114*	0.0687	0.0112	*KCNE1*	0.0176	0.0609
BRWD1	0.1014	0.0468	*ZNF295-AS1*	0.0687	0.0112	*COL6A2*	0.0176	0.1059
IL10RB-AS1	0.1012	0.0211	*LINC00313*	0.0687	0.0112	*CLIC6*	0.0160	0.1100
CBR3	0.1004	0.0667	*DIP2A-IT1*	0.0687	0.0112	*TFF3*	0.0156	0.0483
LOC101928796	0.0997	0.0104	*HUNK*	0.0687	0.0112	*PCBP3*	0.0130	0.0710
PKNOX1	0.0984	0.0302	*FTCD*	0.0675	0.0777	*LINC00649*	0.0124	0.0922
SYNJ1	0.0982	0.0925	*PAXBP1-AS1*	0.0668	0.0637	*CBS*	0.0116	0.0428
USP25	0.0978	0.0398	*RUNX1*	0.0664	0.0679	*ITGB2*	0.0105	0.1184
DIP2A	0.0965	0.0806	*MIS18A*	0.0662	0.1012	*COL6A1*	0.0105	0.1586
NDUFV3	0.0959	0.0084	*SLC5A3*	0.0654	0.1170	*LINC00479*	0.0100	0.1398
TCP10L	0.0958	0.0215	*MAP3K7CL*	0.0654	0.0751	*KRTAP20-2*	0.0094	0.0813
SETD4	0.0951	0.0412	*LINC00310*	0.0640	0.0501	*PCP4*	0.0093	0.0128
CSTB	0.0947	0.0759	*CYYR1*	0.0631	0.0130	*IGSF5*	0.0089	0.0809
GABPA	0.0946	0.0924	*ABCG1*	0.0629	0.0775	*ITGB2-AS1*	0.0074	0.0455
S100B	0.0940	0.0379	*RWDD2B*	0.0607	0.0232	*TMPRSS2*	0.0060	0.1166
CRYZL1	0.0930	0.0818	*C2CD2*	0.0602	0.0106	*TRPM2*	0.0059	0.0483
LTN1	0.0925	0.0736	*HSPA13*	0.0586	0.0409	*TPTE*	0.0053	0.0455
TIAM1	0.0921	0.0849	*PRDM15*	0.0570	0.0676	*LINC00160*	0.0049	0.0380
IL10RB	0.0916	0.0827	*SUMO3*	0.0567	0.0123	*TMPRSS3*	0.0047	0.0799
TTC3	0.0910	0.1090	*SSR4P1*	0.0548	0.1445	*MIR155HG*	0.0045	0.0776
IFNAR2	0.0908	0.0953	*DOPEY2*	0.0544	0.1216	*CBR1*	0.0045	0.0760
RRP1B	0.0900	0.1140	*ICOSLG*	0.0540	0.0249	*ANKRD30BP2*	0.0045	0.0612
ADAMTS5	0.0897	0.0387	*LINC00322*	0.0521	0.1401	*GRIK1-AS1*	0.0036	0.0768
PAXBP1	0.0896	0.0933	*ZBTB21*	0.0511	0.1283	*KCNJ15*	0.0025	0.0231
IFNAR1	0.0895	0.0105	*ADAMTS1*	0.0505	0.1181	*RBM11*	0.0012	0.0418
HMGN1	0.0868	0.1171	*SIM2*	0.0501	0.0424	*BAGE2*	0.0003	0.0115
MCM3AP	0.0867	0.0088	*POFUT2*	0.0489	0.1077			
MRPS6	0.0863	0.1074	*CXADR*	0.0432	0.1145			

Absolute values from the PCA of 178 genes in chromosome 21 showing positive read counts, in at least one of the APC lines, based on RNA-seq data.

Supplementary Table 4. sgRNA sequences for CRISPR–Cas9.

Purpose	Target site	Sequence
XIST insertion	*DYRK1A* intron 1	AGTTTACACGGGTGATGAGC
DYRK1A targeting	*DYRK1A* intron 8	GATGGAAAACGGGTAAAATA

Supplementary Table 5. Primers for *DYRK1A* targeting.

Purpose	Forward primer (5' to 3')	Reverse primer (5' to 3')
RT-PCR (exon 8–10)	F1; TGACCAAGCACCAAAAGCAAG	R2; CGGGTTCCTGGTGGTTTGTA
5' Junctional PCR	F2; CACGCCACTGCCCTCTAGCTTGGGAGACAG	R3; GGCCGATGCAAAGTGCCGATAAACATAACG
3' Junctional PCR	F3; TTGACGGCAATTTCGATGATGCAGCTTGGG	R2; CTGGTGGCAGCGTCCGCTAAAATACAAGGC
Outside PCR	F2; CACGCCACTGCCCTCTAGCTTGGGAGACAG	R2; CTGGTGGCAGCGTCCGCTAAAATACAAGGC
STR	GCACCCAACCCAGTTTTCAGAG (Fluor)	GCTGTCTTCCCACTATAACATAAAC

Supplementary Table 6. Primers for Quantitative RT-PCR.

Gene	Forward primer (5' to 3')	Reverse primer (5' to 3')
ACTB	TCAAGATCATTGCTCCTCCTGAG	ACATCTGCTGGAAGGTGGACA
XIST	AGCTCCTCGGACAGCTGTAA	GGACACATGCAGCGTGGTA
CCT8	TTTTTCCTGATTCCGGCCATT	AACCATGCCATGCAATACTGA
RCAN1	GCTCCGCCAAATCCAGACAA	GCTGCGTGCAATTCATACTTTTC
DYRK1A	ACCACCTCCAGTACATCGTCAAG	CCGGGCTCTCCCACTGTT
C21orf33	GATGCGGAGTCTACGATGGG	CCAGGTCTGTGATTTTGCCAC
rtTA	CTGGGAGTTGAGCAGCCTAC	TCCAGCATCTCGATTGGCAG
DSCR3	CCTACACTGGAGACCACCAACTT	GATGAGGTGGTCAGGGTGAAG
PIGP	TGTTTGGGATTAACATGATGAGTACCT	TCTGCTGTTGATTTTTTGCATAGTT
HLCS	AGGACAAAGGGCCCAACAG	CGCTGCCCAGATGGACTT

Supplementary Table 7. Primers for allele-specific SNP analysis.

Purpose	Forward primer (5' to 3')	Reverse primer (5' to 3')
PCR for allele-specific SNP analysis	GCATGAATGGCCAGATGCTG	CAATCCTGCCTTTCCTGGGT
Sanger sequencing	TTGTGGGTGACATTCTCTGG	-
