# Supplementary material for: A human isogenic iPSC-derived cell line panel identifies major regulators of aberrant astrocyte proliferation in Down syndrome
Source: Commun Biol. 2021 Jun 14;4:730. doi: 10.1038/s42003-021-02242-7 (PMC8203796; doi:10.1038/s42003-021-02242-7)
Supplement: Supplementary file 3 — Descriptions of Additional Supplementary Files [file 42003_2021_2242_MOESM3_ESM.pdf]

Descriptions of Additional Supplementary Files

Supplementary data 1
Description: Relative expression levels of the genes on chromosome 21. Genes are listed according to chromosomal position. Expression was normalized to that of cDi21 lines (n = 3 experiments per cell line). Data were analysed by Welch's two-sample t-test.

Supplementary data 2
Description: The enlarged heat map related to Figure 3b.

Supplementary data 3
Description: Source data underlying all figures.
