# Supplementary material for: A human isogenic iPSC-derived cell line panel identifies major regulators of aberrant astrocyte proliferation in Down syndrome
Source: Commun Biol. 2021 Jun 14;4:730. doi: 10.1038/s42003-021-02242-7 (PMC8203796; doi:10.1038/s42003-021-02242-7)
Supplement: Supplementary file 7 — Reporting Summary [file 42003_2021_2242_MOESM7_ESM.pdf]

nature research

Corresponding author(s): Yasuji Kitabatake

Last updated by author(s): Apr 19, 2021

Reporting Summary

Nature Research wishes to improve the reproducibility of the work that we publish. This form provides structure for consistency and transparency in reporting. For further information on Nature Research policies, see our Editorial Policies and the Editorial Policy Checklist.

Statistics

For all statistical analyses, confirm that the following items are present in the figure legend, table legend, main text, or Methods section.

n/a	Confirmed	
☐	☒	The exact sample size (*n*) for each experimental group/condition, given as a discrete number and unit of measurement
☐	☒	A statement on whether measurements were taken from distinct samples or whether the same sample was measured repeatedly
☐	☒	The statistical test(s) used AND whether they are one- or two-sided<br>*Only common tests should be described solely by name; describe more complex techniques in the Methods section.*
☒	☐	A description of all covariates tested
☐	☒	A description of any assumptions or corrections, such as tests of normality and adjustment for multiple comparisons
☒	☐	A full description of the statistical parameters including central tendency (e.g. means) or other basic estimates (e.g. regression coefficient) AND variation (e.g. standard deviation) or associated estimates of uncertainty (e.g. confidence intervals)
☒	☐	For null hypothesis testing, the test statistic (e.g. *F*, *t*, *r*) with confidence intervals, effect sizes, degrees of freedom and *P* value noted<br>*Give P values as exact values whenever suitable.*
☒	☐	For Bayesian analysis, information on the choice of priors and Markov chain Monte Carlo settings
☐	☒	For hierarchical and complex designs, identification of the appropriate level for tests and full reporting of outcomes
☒	☐	Estimates of effect sizes (e.g. Cohen's *d*, Pearson's *r*), indicating how they were calculated

Our web collection on statistics for biologists contains articles on many of the points above.

Software and code

Policy information about availability of computer code

Data collection	No software was used to collect data.
Data analysis	Immunocytochemistry: In Cell Developer Toolbox 1.9 (GE Healthcare).<br>Western blotting: ImageJ 1.51j8 (http://imagej.nih.gov/ij/).<br>Statistical analysis: EZR 1.52 (http://www.jichi.ac.jp/saitama-sct/SaitamaHP.files/statmed.html)<br>RNA-seq: TopHat 2.1.1, bowtie2 2.3.2, StrandNGS 3.2(Agilent Technologies) and ggplot2 3.3.2, R 3.6.<br>ChIP-seq: Picard 1.119, MEDIPS 1.30 and R 3.4.

For manuscripts utilizing custom algorithms or software that are central to the research but not yet described in published literature, software must be made available to editors and reviewers. We strongly encourage code deposition in a community repository (e.g. GitHub). See the Nature Research guidelines for submitting code & software for further information.

Data

Policy information about availability of data

All manuscripts must include a data availability statement. This statement should provide the following information, where applicable:
- Accession codes, unique identifiers, or web links for publicly available datasets
- A list of figures that have associated raw data
- A description of any restrictions on data availability

The RNA-seq data and ChIP-seq data are available in the DDBJ Sequenced Archive under accession numbers DRA010528 (https://ddbj.nig.ac.jp/DRASearch/submission?acc=DRA010528) and DRA010529 (https://ddbj.nig.ac.jp/DRASearch/submission?acc=DRA010529), respectively.

Field-specific reporting

Please select the one below that is the best fit for your research. If you are not sure, read the appropriate sections before making your selection.

[x] Life sciences   [ ] Behavioural & social sciences   [ ] Ecological, evolutionary & environmental sciences

For a reference copy of the document with all sections, see nature.com/documents/nr-reporting-summary-flat.pdf

Life sciences study design

All studies must disclose on these points even when the disclosure is negative.

Sample size	Sample size calculations were not performed. Sample size was determined depending on the experiment type based on what is standard practice in the field of cell biology.
Data exclusions	No data was excluded from this analysis.
Replication	All experiments were successfully reproduced, and the number of replications is indicated in the Figure Legends.
Randomization	No randomization was done because the data were processed though a standardized analysis that excluded human bias.
Blinding	This study design did not contain a comparison between lines that needs to consider blinding.

Reporting for specific materials, systems and methods

We require information from authors about some types of materials, experimental systems and methods used in many studies. Here, indicate whether each material, system or method listed is relevant to your study. If you are not sure if a list item applies to your research, read the appropriate section before selecting a response.

Materials & experimental systems

n/a	Involved in the study	
[ ]	[x]	Antibodies
[ ]	[x]	Eukaryotic cell lines
[x]	[ ]	Palaeontology and archaeology
[x]	[ ]	Animals and other organisms
[ ]	[x]	Human research participants
[x]	[ ]	Clinical data
[x]	[ ]	Dual use research of concern

Methods

n/a	Involved in the study	
[ ]	[x]	ChIP-seq
[x]	[ ]	Flow cytometry
[x]	[ ]	MRI-based neuroimaging

Antibodies

Antibodies used	For immunocytochemistry, anti-OCT4 (Santa Cruz Biotechnology, sc-5279, 1:200), anti-SSEA4 (Merck, MAB4304, 1:200), anti-H3K27me3 (Merck, 07-449, 1:200), anti-PAX6 (Stemgent, 09-0075, 1:100), anti-SOX1 (R&D Systems, AF3369, 1:100), anti-GFAP (DakoCytomation, Z0334, 1:1000), anti-S100β (Merck, S2532, 1:1000), anti-CD44 (Merck, MABF580, 1:200)and anti-vimentin (Merck, V2258, 1:500) antibodies were used.<br>For western blotting, anti-STAT3 (Cell Signaling Technology, 9139, 1:1000), anti-Phospho-STAT3 (Ser727) (Cell Signaling Technology, 9134, 1:500) , anti-DYRK1A (Cell Signaling Technology, 2771, 1:1000), anti-Cyclin D1 (Cell Signaling Technology, 55506, 1:1000), anti-p27KIP1 (Cell Signaling Technology, 3686, 1:1000), anti-p21CIP1 (Cell Signaling Technology, 2947, 1:1000) and anti-β-actin (MBL, PM053-7, 1:2000) antibodies were used.
Validation	Antibodies used in this study have been validated by the suppliers for the applications for which they were used, and have been cited in multiple publications.

Eukaryotic cell lines

Policy information about cell lines

Cell line source(s)	iPSCs are derived from cord blood mononuclear cells of a male baby with Down syndrome in Osaka University Hospital, Osaka, Japan. I conducted experiments to use astrocyte precursor cells and neural precursor cells which were differentiated from the iPSCs.
Authentication	Cell lines were generated in house and were not authenticated.
Mycoplasma contamination	Cell lines have been tested negative for Mycoplasma contamination.

Commonly misidentified lines (See ICLAC register)	No commonly misidentified lines were used in this study.

Human research participants

Policy information about studies involving human research participants

Population characteristics	We focused on babies who were diagnosed with Down syndrome before birth in Osaka University Hospital, Osaka, Japan.
Recruitment	We explained about this study to the baby's parents before and after birth. After informed consent was obtained from them in accordance with the Declaration of Helsinki, we obtained cord blood mononuclear cells at birth to induce to iPSCs.
Ethics oversight	This study was approved by the Ethics Committee of Osaka University, Osaka, Japan.

Note that full information on the approval of the study protocol must also be provided in the manuscript.

ChIP-seq

Data deposition

- [x] Confirm that both raw and final processed data have been deposited in a public database such as GEO.
- [ ] Confirm that you have deposited or provided access to graph files (e.g. BED files) for the called peaks.

Data access links *May remain private before publication.*	ChIP-seq data are available in the DDBJ Sequenced Archive under accession number DRA010529 (https://ddbj.nig.ac.jp/DRASearch/submission?acc=DRA010529).
Files in database submission	1_APC (Dox−)_1_R1.fastq.gz<br>2_APC (Dox−)_2_R1.fastq.gz<br>3_APC (Dox−)_3_R1.fastq.gz<br>4_APC (Dox+)_1_R1.fastq.gz<br>5_APC (Dox+)_2_R1.fastq.gz<br>6_APC (Dox+)_3_R1.fastq.gz<br>7_APC (Dox_remov)_1_R1.fastq.gz<br>8_APC (Dox_remov)_2_R1.fastq.gz<br>9_APC (Dox_remov)_3_R1.fastq.gz<br>10_APC (corrected_disomy)_1_R1.fastq.gz<br>11_APC (corrected_disomy)_2_R1.fastq.gz<br>12_APC (corrected_disomy)_3_R1.fastq.gz<br>13_APC (input)_1_R1.fastq.gz<br>14_APC (input)_2_R1.fastq.gz<br>15_APC (input)_3_R1.fastq.gz
Genome browser session (e.g. UCSC)	N/A

Methodology

Replicates	Three biological replicates for each cell line.
Sequencing depth	sample name, total reads, unique mapped reads, read length, read type<br>APC (Dox−)_1, 28135640, 26236461, 75, single-end<br>APC (Dox−)_2, 31921935, 29614185, 75, single-end<br>APC (Dox−)_3, 26466818, 24676631, 75, single-end<br>APC (Dox+)_1, 28376684, 26356595, 75, single-end<br>APC (Dox+)_2, 30232295, 28113442, 75, single-end<br>APC (Dox+)_3, 31310890, 29044766, 75, single-end<br>APC (Dox_remov)_1, 26979830, 25085964, 75, single-end<br>APC (Dox_remov)_2, 27769610, 25834873, 75, single-end<br>APC (Dox_remov)_3, 32574203, 30193378, 75, single-end<br>APC (corrected_disomy)_1, 29934067, 27795098, 75, single-end<br>APC (corrected_disomy)_2, 31451501, 29210827, 75, single-end<br>APC (corrected_disomy)_3, 28253777, 26302776, 75, single-end<br>APC (input)_1, 34748645, 31819415, 75, single-end<br>APC (input)_2, 37321435, 34321834, 75, single-end<br>APC (input)_3, 33038833, 30708721, 75, single-end
Antibodies	Anti-H3K27me3 antibody (Cell Signaling Technology, 9733) was used for ChIP.
Peak calling parameters	N/A

Data quality	Reads were quality controlled using FastQC. Reads with a mapping quality score > 20 were retained for this study. Coverage was calculated with genome-wide 100-bp bins, with read depth more than 10 using MEDIPS software.
Software	Picard 1.119 and MEDIPS 1.30, R 3.4.
